# Automatic Estimation of Coronary Blood Flow Velocity Step 1 for Developing a Tool to Diagnose Patients With Micro-Vascular Angina Pectoris

**DOI:** 10.3389/fcvm.2019.00001

**Published:** 2019-01-22

**Authors:** Mahdieh Khanmohammadi, Kjersti Engan, Charlotte Sæland, Trygve Eftestøl, Alf I. Larsen

**Affiliations:** ^1^University of Stavanger Department of Electrical Engineering and Computer Science, Stavanger, Norway; ^2^Stavanger University Hospital Department of Cardiology, Stavanger, Norway; ^3^Department of Clinical Science, University of Bergen, Bergen, Norway

**Keywords:** blood flow velocity, X-ray angiography, image registration, automatic segmentation, transthoracic doppler, coronary arteries, coronary flow reserve

## Abstract

**Aim:** Our aim was to automatically estimate the blood velocity in coronary arteries using cine X-ray angiographic sequence. Estimating the coronary blood velocity is a key approach in investigating patients with angina pectoris and no significant coronary artery disease. Blood velocity estimation is central in assessing coronary flow reserve.

**Methods and Results:** A multi-step automatic method for blood flow velocity estimation based on the information extracted solely from the cine X-ray coronary angiography sequence obtained by invasive selective coronary catheterization was developed. The method includes (1) an iterative process of segmenting coronary arteries modeling and removing the heart motion using a non-rigid registration, (2) measuring the area of the segmented arteries in each frame, (3) fitting the measured sequence of areas with a 7° polynomial to find start and stop time of dye propagation, and (4) estimating the blood flow velocity based on the time of the dye propagation and the length of the artery-tree. To evaluate the method, coronary angiography recordings from 21 patients with no obstructive coronary artery disease were used. In addition, coronary flow velocity was measured in the same patients using a modified transthoracic Doppler assessment of the left anterior descending artery. We found a moderate but statistically significant correlation between flow velocity assessed by trans thoracic Doppler and the proposed method applying both Spearman and Pearson tests.

**Conclusion:** Measures of coronary flow velocity using a novel fully automatic method that utilizes the information from the X-ray coronary angiographic sequence were statistically significantly correlated to measurements obtained with transthoracic Doppler recordings.

## Introduction

Myocardial ischemia is due to an imbalance between myocardial metabolic demand and coronary blood supply. This is mainly related to epicardial atherosclerotic coronary artery disease (CAD) (1). However, the angiographic evidence of a “normal” or mildly diseased epicardial coronary tree, usually defined as the absence of a luminal diameter reduction of ≥50% (or >70% of the luminal area reduction) (2), is a common finding, as it is documented in ~25% of patients undergoing coronary angiography (3). This condition is usually defined as angina with “normal” coronary arteries or, more correctly, angina in the absence of obstructive CAD.

Psychological morbidity with great impact on daily living is well known in both patients with cardiovascular disease and in patients with angina in the absence of obstructive CAD. These patients constitute a therapeutic problem with considerable residual morbidity associated with functional limitation and reduced quality of life (4). In addition, a relatively large proportion of these patients are taken care of by the health authority system indicating that this issue has economic consequences for the society that is not negligible.

Our aim was to estimate the blood velocity in coronary arteries using a novel automatical algorithm employing X-ray angiographic sequence obtained by selective invasive coronary catheterization. Blood velocity can later be used to assess coronary flow reserve (CFR) by estimating the ratio between blood flow during full hyperemia using adenosine infusion and blood flow velocity at rest. Impaired CFR is associated with increased morbidity and mortality in this population (3, 5).

Several methods have been utilized to indirectly assess micro vascular function including intracoronary Doppler measurements. CFR can now be calculated using transthoracic Doppler registration that makes it independent of an invasive procedure (6). Non-invasive measurement of coronary flow velocity (CFV) and coronary flow velocity reserve (CFVR) in the distal left anterior descending artery (LAD) using transthoracic Doppler echocardiography (TTDE) accurately reflects invasive measurement of CFV and CFVR by Doppler guide wire (DGW) method (7, 8).

An automatic method of estimating CFR using non-invasive techniques has been of great interests for the cardiology society for years and recently new techniques have been developed to assess the coronary flow reserve by means of positron emission tomography (PET) (9–11); contrast stress echocardiography and cardiac magnetic resonance (CMR) imaging (2, 12). Moreover, blood velocity has been calculated from volumetric dynamic computed tomography angiography (13). In addition, arterial flow has been quantified by using 3-dimensional (3D) rotational X-ray angiography (14). However, the use of imaging modalities is limited due to excessive costs and inaccessibility in small hospitals. Moreover, some of the described techniques are computationally heavy and time consuming. On the contrary, cine X-ray coronary angiography sequences obtained by invasive coronary catheterization is a routine imaging procedure normally to assess suspected coronary artery disease in patients with documented ischemia or classical symptoms of angina pectoris. Despite the availability of numerous non-invasive tests for the detection of coronary-artery stenoses, coronary angiography remains the common diagnostic procedure for stenosis evaluation with the immediate possibility to perform percutaneous coronary intervention if necessary (15).

Thus, the ability to estimate the *blood velocity* in coronary arteries by only using the coronary angiography sequence can form the fundamental basics for developing an alternative method for assessing CFR without using intracoronary Doppler wires during the first standard invasive angiography.

The goal for the current study was to develop a mathematical model to automatically estimate how fast blood propagates in coronary arteries using X-ray coronary angiographic sequences and to compare these estimates with transthoracic Doppler measurements of coronary flow velocity in patients with chest pain and normal coronary arteries (CPNCA).

## Methods

### Patient Enrollment

Patients with a history of repeated episodes of exercise induced chest pain and normal or near normal coronary angiography were screened for inclusion in the “The Syndrome X-ercise study (SYNDEX)”; clinicaltrials.gov # identifier: NCT02905630, at the department of cardiology, Stavanger University Hospital. The patients had to be of 18 years or older and being able to participate in training groups 3 times a week. Patients were excluded if they had other serious cardiac illness, cancer or contrast agent allergy. Twenty-one patients were included in the study. The initial aim of the study was to identify possible effects of high intensity exercise training on coronary flow reserve and its relationship to experienced angina In addition peak oxygen consumption (peak VO_2_) measured with breath-to-breath ergospirometry (during a graded treadmill exercise test); and endothelial function were assessed. All the patients signed informed consent form. This study was carried out in accordance with the recommendations of the Helsinki declaration (2013/98-8), Norwegian Regional Committee for Medical and Health Research Ethics. The protocol was approved by the Norwegian Regional Committee for Medical and Health Research Ethics. All subjects gave written informed consent in accordance with the Declaration of Helsinki.

### Image Acquisition

For all patients, cine X-ray Coronary angiography sequences were obtained by invasive coronary catheterization. Later in the manuscript this is simplified to coronary angiography, and the time-sequence of images as angiographic sequence. Standard selective coronary artery angiography with 6 Fr catheters using a GE coronary angio-laboratory and X-ray contrast medium (Iomeron 350) was performed. Manual injection of contrast agent with an approximate flow rate of 1 to 2 cc/s not exceeding 10 mL for each view was performed in standard views. A 10 cc syringe was used by a well-trained interventional cardiologist during selective coronary catheterization to do injection of contrast agent with an approximate flow rate of 1 to 2 cc/s for each standard view. All patients had normal coronary arteries with no proximal stenosis that would make selective catheterization difficult. All angles used for angiography and height of table above the radiation source were recorded. The sequences were acquired at 15 frames per second, with a pixel resolution of 0.2 mm per pixel and a bit-depth of 8 bits per pixel.

Coronary flow velocity was measured using a modified transthoracic Doppler at the mid part of the left anterior descending artery (LAD) in accordance with current standards (6). Patients were examined using GE ultrasound systems, Vivid 5, Horten Norway, with coronary flow probe, without using a contrast agent. The velocity was mainly measured in the distal to the mid left anterior descending (LAD) coronary artery. Alternatively, flow velocities were measured in marginal branches from the left circumflex coronary artery (CMB) or posterior descending coronary artery (PDA) if flow velocities in the LAD could not be satisfactorily measured.

Blood flow velocities were measured using pulsed-wave Doppler with 1.75 to 3.5 MHz frequencies.

### Proposed Technical Method

**Figure 1:**
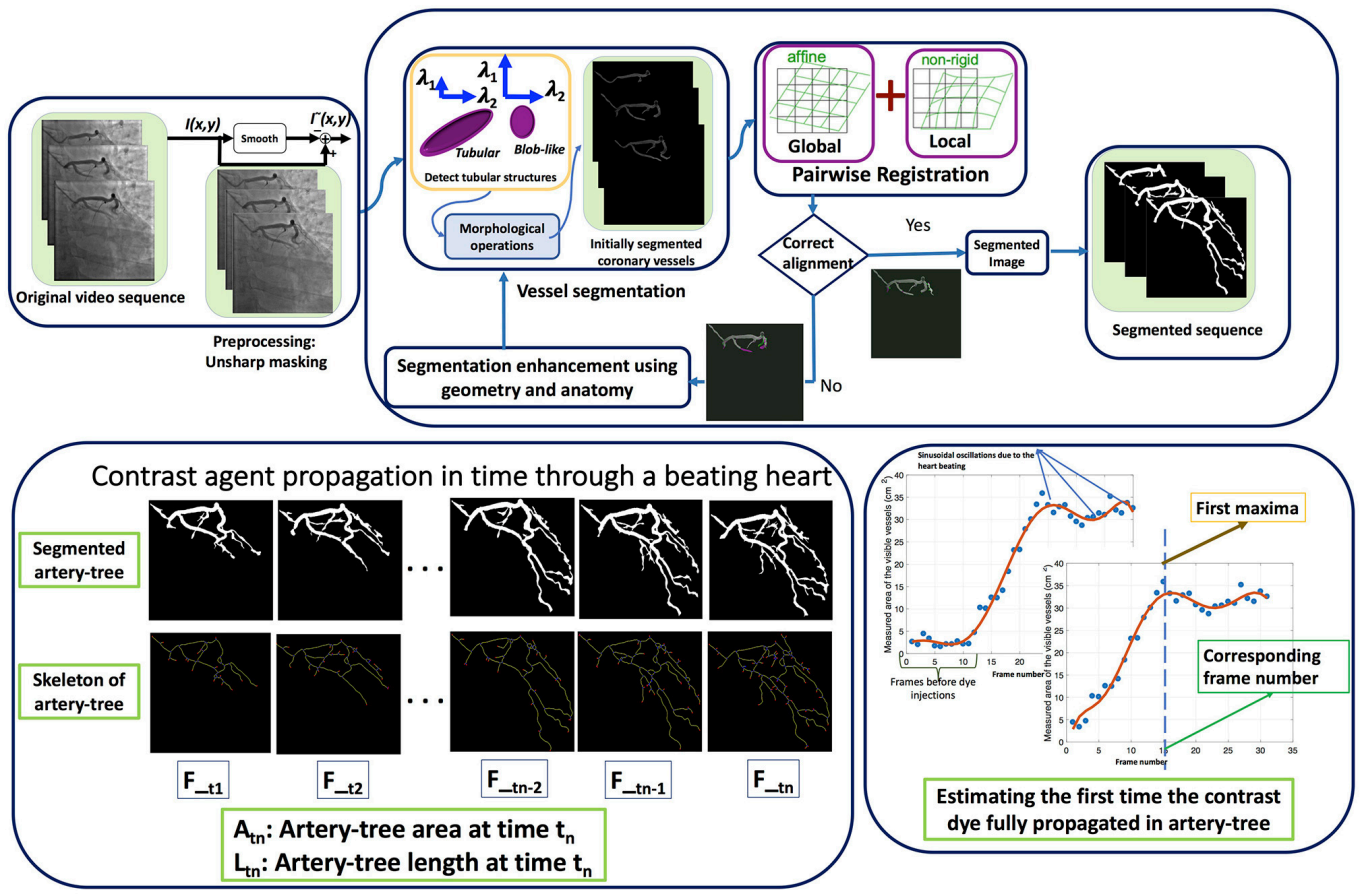
The flowchart of the algorithm. The first row shows different steps of the automatic artery-tree segmentation and the second row shows the segmentation results in some sample frames of a video sequence along with the skeleton of the artery-tree. The last part shows how the required time for contrast dye full propagation through the artery-tree is estimated.

In this work, we propose a method for estimating the blood velocity utilizing the movement of the contrast fluid as it fills the coronary arteries during invasively obtained angiographic sequences. A robust and accurate automatic segmentation technique for the coronary arteries during dye propagation is required and we use an upgraded version of our recently developed method (16), presented in the following:

First the artifact of other chest cavity organs present in the images are suppressed and the edges of the arteries are sharpened by preprocessing. Thereafter, the first segmentation is done using Frangi vesselness Hessian filters (17–19), followed by morphological operations. Furthermore, the heart motion is modeled and removed by employing a non-rigid spline registration technique on the segmented vessel images. The aligned images are evaluated to determine whether the vessel segmentation is satisfactory. We propose an iterative method that finds the misaligned segmented images, then enhances the segmentations of both images, using anatomical and geometrical information of the coronary arteries, and updates the input of the registration step. The changes in the length of the segmented artery-tree in time give an estimation of the velocity of the propagation of the contrast fluid through the coronary arteries. Based on this we estimate the velocity of the blood in angiograms captured from coronary arteries. A flow chart illustrating the overview of the proposed method is shown in Figure 1. In the following, more details are provided on the different parts of the proposed method.

### Preprocessing

The imaging method introduces noise and artifacts, which must be reliably suppressed or avoided to gain consistent information. The observed noise and artifact sources comprise of (1) the shadow of other present organs in the chest cavity (bones and lungs), (2) the heart motion that complicates the contrast agent movements, and (3) movements due to breathing, (4) appearance of external objects in some of the images for some patients.

An unsharp masking technique is used to sharpen the edges of the coronary vessels and to suppress the shadow of other organs than heart artery-tree (20). The unsharp contrast enhancement filter used in this study is of size 3-by-3 from the negative of the Laplacian filter. Applying this filter to the original image produce an estimation of a blurred version of the image. Then this estimation is subtracted from the original image, resulting in a sharp image with enhanced edges. Let *I*^{*k*}^ be the *k*th image frame of the sequence, then the sharp image is estimated using the following equation: ${I_{s}}^{\left\{k\right\}}$ = *I*^{*k*}^ − $\left(I^{\left\{k\right\}}*f_{laplace}\right)$, where ^*^ denotes convolution.

### Initial Segmentation of the Coronary Artery Tree

The coronary arteries, as all blood vessels, have shape as tubes or pipes in the angiograms, and they appear dark in the X-ray angiographic sequences. The first step of the vessel segmentation is to exploit this knowledge. An image processing filter technique is utilized to emphasize such shapes, using 2D Frangi vesselness Hessian filter (17), to initially detect vessels in each angiography video frame independently. The Frangi vesselness filter is well used and studied, and examples and details can be found in the sample references (18, 19). The method outputs an image frame, *I*_*f*_, where the pixels are interpreted as probabilities for the corresponding pixel of the input image frame to be part of a vessel or not. Subsequently the probabilities given in *I*_*f*_ are thresholded with a parameter *th*, generating a binary image with potential vessels as segments. After keeping the segmented areas corresponding to the dark tubular structures in the image sequence, morphological image processing operations such as opening and closing were applied to detect the largest component representing the main coronary artery-tree in the image. The resulting segmented frames compromise this initial segmented coronary artery tree found from each frame independently from the previous or next in time.

### Removing Heart Motion Using Non-rigid Registration

Using Frangi vesselness filter and 2D image information gives a first approximation to the segmentation of the artery-tree frame by frame. However, with use of the image sequence, the similarity between consecutive frames can be utilized to improve the segmentation. The changes in the shape and the length of the visible artery-tree in the image sequence are due to two major factors, (1) the beating of the heart; contractions and expansions; (2) the length of visible artery-tree increasing as the dye propagates further into the arteries. If the heart motion can be modeled and removed from the image sequence the only strong motion of interest is of the contrast agent moving through the arteries.

The beating heart has a non-rigid motion, which can be well modeled by an affine transformation plus a free-form deformation (FFD) based on B-spline. Therefore, we tailored the algorithm developed by Rueckert et al. (21), which combines global and local transformations providing a high degree of flexibility to model a 3D deformable object. Spline-based FFD has also shown good results in tracking and analyzing the motion of cardiac images using positron emission tomography (22). The global motion describes the overall motion of the heart that we model with affine transformation, a general class of transformations with 12° of freedom to describe rotation, translation, scaling, and shearing. In addition, FFD was used to model the local deformation of the heart and consequently the artery-tree. The local deformations have variable nature and can vary between patients and with age, and are not well modeled through parameterized transformations.

As a step of registration, a similarity measure is required to relate the two images and measure the degree of alignment between them. If the two images are aligned the similarity measure is maximized. Usually a direct comparison of image intensities, for example sum of squared differences or correlation, is used to measure the similarity between the two images. However, the propagation of the contrast agent during the sequence induces a change in image intensities from frame to frame. Thus, a direct comparison of image intensities is not an accurate similarity measure for this application. Therefore, normalized mutual information was used as a similarity criterion, which is based on information theory and expresses the amount of information that one of the images contains about the other (23, 24).

### Enhancing the Segmented Artery-Tree Based on Registration Results

The inputs to the registration step are binary frames corresponding to the segmented artery-tree. Thus, the accuracy of the registration is dependent on the quality of the segmentations. Missing parts of the artery-tree in the segmentations from one frame to the next can either be the result of poor segmentation or contrast agent propagation over the time. However, we assume that the vessel shape changes due to the propagation of the contrast agent are considerably smaller compared to the disturbances caused by the heart movement in the two consecutive images. Therefore, an adequately large misalignment in a pair of frames after registration is assumedly caused by poor segmentation.

If poor segmentation is detected, the probability output from the Frangi filter, *I*_*f*_, is thresholded again with a lower threshold *th*. This will typically include more segmented areas, and the additional areas are examined to justify if they should be included to the artery-tree segmentation or not. This decision is based on their distance to the main artery-tree, both the distance to center and the edge of the previously segmented area. After enhancing the segmentations, the segmented images were saved into the sequence and underwent another round of registration. This procedure was repeated until the registration results were satisfactory. This satisfaction factor was defined based on the difference in number of pixels in the two aligned images. For example, the number of segmented pixels in frame number two is always larger than number of segmented pixels in frame number one due to the propagation of the dye, at the same time the difference is not expected to be very large, since it should only correspond to the dye movement. Therefore, the satisfactory factor was defined so that the $\underset{all\;pixels\;}{\sum }\left(B^{\left\{k\right\}}-B^{\left\{k-1\right\}}\right)\;\in \left\{500,\;1500\right\}$ pixels, where *B*^{*k*}^ is the k-th binary image in the sequence, after segmentation and registration, with ones at the position of the segmented arteries. The limits were chosen empirically.

### Velocity Estimation

In hemodynamics it is commonly assumed that in medium to large arteries, the blood can be modeled as an incompressible Newtonian fluid. The coronary arteries are considered to be medium sized arteries. The flowrate, Q, of an incompressible fluid is considered constant, even if the cross sectional area, A, is changing, or if the pipe is branching. This gives the relationship *Q* = *A*_1_·*v*_1_ = *A*_2_·*v*_2_ for a single pipe and *Q* = *n*_1_·*A*_1_·*v*_1_ =_*n*_2_·*A*2_·*v*_2_ for branching pipes (or arteries) when assuming that the cross section over the different branches are all *A*_1_ before the branching and *A*_2_ after the branching. *n*_*i*_ denotes the number of branches, and *v*_*i*_ denotes the average velocity over the cross section. The velocity is defined as the length that a blood particle has moved over a period of time, $v=\frac{\Delta L}{\Delta t}$. The angiographic sequence is acquired at a frame rate of 15 frames per second (fps). The changes from one frame to the next corresponds to a Δt = 1/15 = 0.066 s. In this work some simplifications are done to estimate the blood velocity from the propagating edge of the contrast agent.

#### Assumption (Ass1)

We assume that all vessels in the area of interest, i.e., medium coronary arteries, are of constant Areal, *A*_*i*_ = *A*. This is a limitation because the vessels become thinner down in the branches, but we are not concerned with the smallest arteries and capillaries. This assumption gives us: *n*_1_·*v*_1_ =_*n*_2_·*v*2_ after a branch, and consequently (Equation 1):

$$\begin{array}{l} v_{2}=v_{1}\frac{n_{1}}{n_{2}} \end{array}$$

Let the time *t*_1_ correspond to the time it takes the blood to travel a distance *L*_1_ with 1 branch, *n*_1_ = 1. After a branching into 2, i.e., *n*_2_ = 2, let the time, *t*_2_, correspond to the time it takes the blood to travel a distance, *L*_2_, in one of the new branches (or *L*_3_ in the other branch, because _*L*_2_ = *L*3_ since *A*_*i*_ = *A*). The total time:

$$\begin{array}{l} {t_{tot}=t}_{1}+t_{2\;}=\frac{L_{1}}{v_{1}}+\frac{L_{2}}{v_{2}} \end{array}$$

From Equation 1 we can say:

$$\begin{array}{l} {t_{tot}=t}_{1}+t_{2\;}=\frac{L_{1}}{v_{1}}+\frac{L_{2}}{v_{1}\;\frac{n_{1}}{n_{2}}}=\frac{L_{1}+n_{2}L_{2}}{v_{1}} \end{array}$$

This gives an estimation for the velocity before the branching as (Equation 2):

$$\begin{array}{l} v_{1}=\frac{L_{1}+2L_{2}}{t_{tot}}=\frac{L_{1}+L_{2}+L_{3}}{t_{tot}} \end{array}$$

From the previous steps, the segmentation of the coronary tree during the propagation of the contrast fluid was found for all image frames throughout the time sequence. A skeletonization of the segmented tree in each frame is now performed to estimate the difference of the length of the arteries from one frame to the next, corresponding to *n*·Δ*L* if all arteries were seen in the tangential direction of the X-ray projection. Of course this is not the case, and this will also impose a limitation of the method, but the angle of the arteries at the edge of the contrast fluid is considered constant from one frame to the next. An estimated velocity can be found as (Equation 3):

$$\begin{array}{l} v_{est}=\frac{{\sum_{i}L}_{i}}{\sum_{j}t_{j}}=\frac{\Delta L_{T}}{\Delta T} \end{array}$$

Where Δ*T* corresponds to a time interval during propagation of the contrast fluid, and Δ*L*_*T*_ corresponds to the total difference of the summed artery length during the time-period Δ*T*, found by the skeletonization of the segmented arteries.

To find the appropriate frames of propagating contrast fluid, we wish to estimate the required time for the contrast agent from the starting time of the injection to full propagation through the coronary arteries, and look at the Δ*L*_*T*_ of the corresponding frames. An angiographic sequence lasts for several heart beat cycles, but full propagation of the contrast agents requires less than a couple of heart cycles.

**Figure 2:**
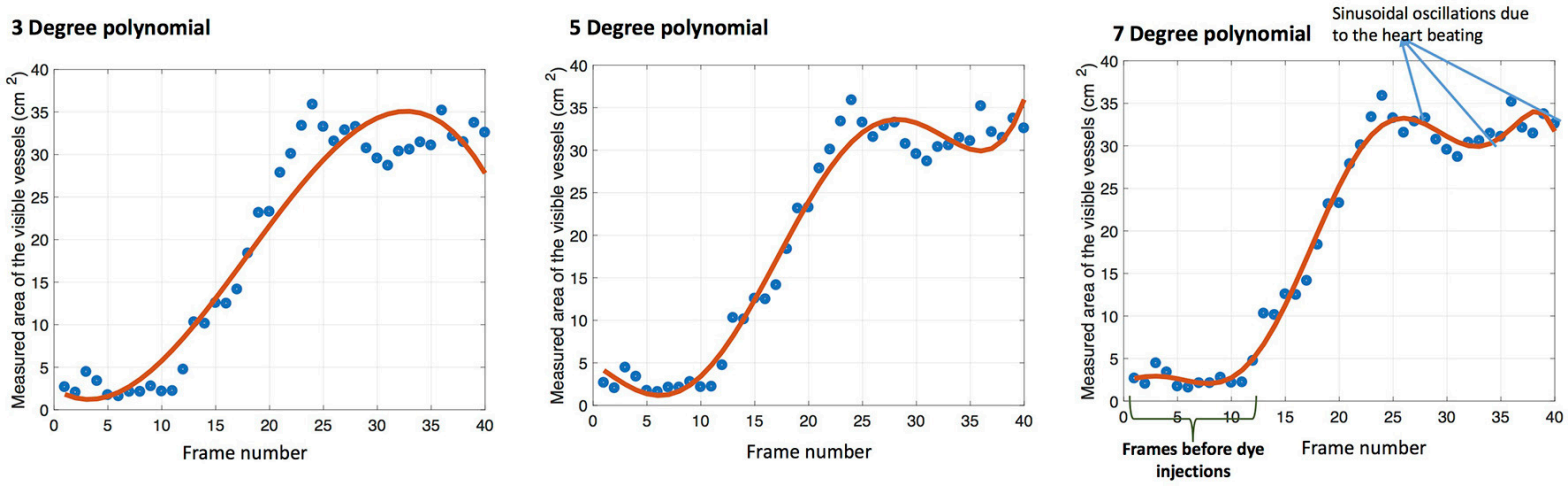
Measured arteries areas in a video sequence is fitted with 3°, 5°, and 7° polynomials from left to right.

The segmented area of the projected artery-tree in XY plane for all frames of the video sequence is found for each patient. It is known that the heart contraction/expansion affects the shape and the size of the coronary artery-tree semi-periodically with the heart cycle. This affects the 2D projections of the artery-tree visible in the angiograms and consequently results in smaller area measures. The change in the shape of the arteries due to the heart motion is rather complex but they have a beneficial characteristic due to the repetitiveness of the heart cycle. Figure 2 shows plots of the coronary artery area measurements, found from the segmented areas, (blue circles) against the frame number for a sample video sequence. This is derived from a sequence from each patient. The difference in area measurements is somewhat noisy, and fitting the data to an appropriate model would be desirable. A polynomial model was chosen due to its simplicity and because it was expected to be able to model the increase of the area during propagation of the dye. This was assumed to be near linear. However, because the heart is moving with every heart beat this gives an overlying fluctuation of the vessel area, caused by the stretching of the vessels rather than the propagation of contrast fluid. A low-order model will not be able to capture the additional fluctuation resulting from the propagation of the fluid, and will typically result in a too low slope angle, illustrated in Figure 2 where 3°, 5°, and 7° polynomial are fitted and displayed with red curves. A seven-order polynomial was chosen as a compromise between accurate modeling of a complex motion and having a simple model based on studying the resulting time sequences. A new and larger dataset should be used to verify this and the remaining part of the proposed method. The time corresponding to the first maxima of the polynomial function is a good estimate of the first time the contrast dye has fully propagated into the segmented part of the coronary artery-tree.

As illustrated in Figure 2, right, some of the captured video frames begin before the contrast dye injection starts, and several frames of the video show only the inserted catheter wire. The start time of the contrast dye injection, *t*_*s*_, and the time of the first maxima of the polynomial function, *t*_*max*_, is found automatically, and the duration between them is Δ*T*. The Δ*L*_*T*_ of the corresponding frames are found from the skelatonization, and the velocity estimated as *v*_*est*_ from Equation 3. The method is denoted *Mlength* in the results, and it provides an estimation of the blood flow velocity in [m/s].

An alternative approach, *Mslope*, is to estimate the slope of the polynomial function as this provides an estimate of the segmented (projected) area over time. Using the same assumption (*Ass1)* were all arteries in the region of interest are considered to have the same cross sectional area, A, the segmented artery-tree area corresponds to the length times the diameter of the arteries. Thus, the slope provides a measure correlated with the blood velocity. The unit is in (pixel area)/(frame time interval), which is equivalent to $\frac{{\left(0.2\;mm\right)}^{2}}{0.066\;s}$. To interpret that as velocity, we would have to estimate the typical diameter of the arteries, but in this work we solely investigate if it is significantly correlated to the Doppler velocities. The 2D projection is imposing an inaccuracy and is thus a limitation.

### Statistical Methods

The estimated velocity should correlate with the measured velocity of the left anterior descending artery from transthoracic Doppler recordings. The Doppler recordings are from the medium sized arteries, not the microvessels, corresponding to the velocity estimated using the proposed method. We evaluated the correlation between coronary flow velocity measured with transthoracic Doppler imaging and the automatic methods, *Mlength* and *Mslope* using Spearman and Pearson tests. This requires normal distribution of the measured velocities either by the proposed technique or from the Doppler recordings, which is tested using one-sample Kolmogorov-Smirnov test (*K*-test).

## Segmentation Assessment

The presented *segmentation algorithm* was applied to 1,428 image frames randomly chosen from 11 patients from each of which the coronary artery-tree was extracted.

Assessing the segmentation algorithm is notoriously hard because of the difficult and time consuming process of manually segmenting the artery-tree. Therefore, we proposed the following alternative method: The cardiologists randomly chose one video sequence per patient from which five images were randomly selected. Then, two types of markers were manually located: (1) artery-tree markers and (2) background markers. The artery-tree markers were put inside the visible arteries and the background markers were put in the close vicinity of the visible arteries. The background markers comprise of higher number of locations in comparison to the artery-tree markers. Totally 300 marker locations were used to construct the manual annotations of the artery-tree and the background in the selected five images of a sequence.

**Figure 3:**
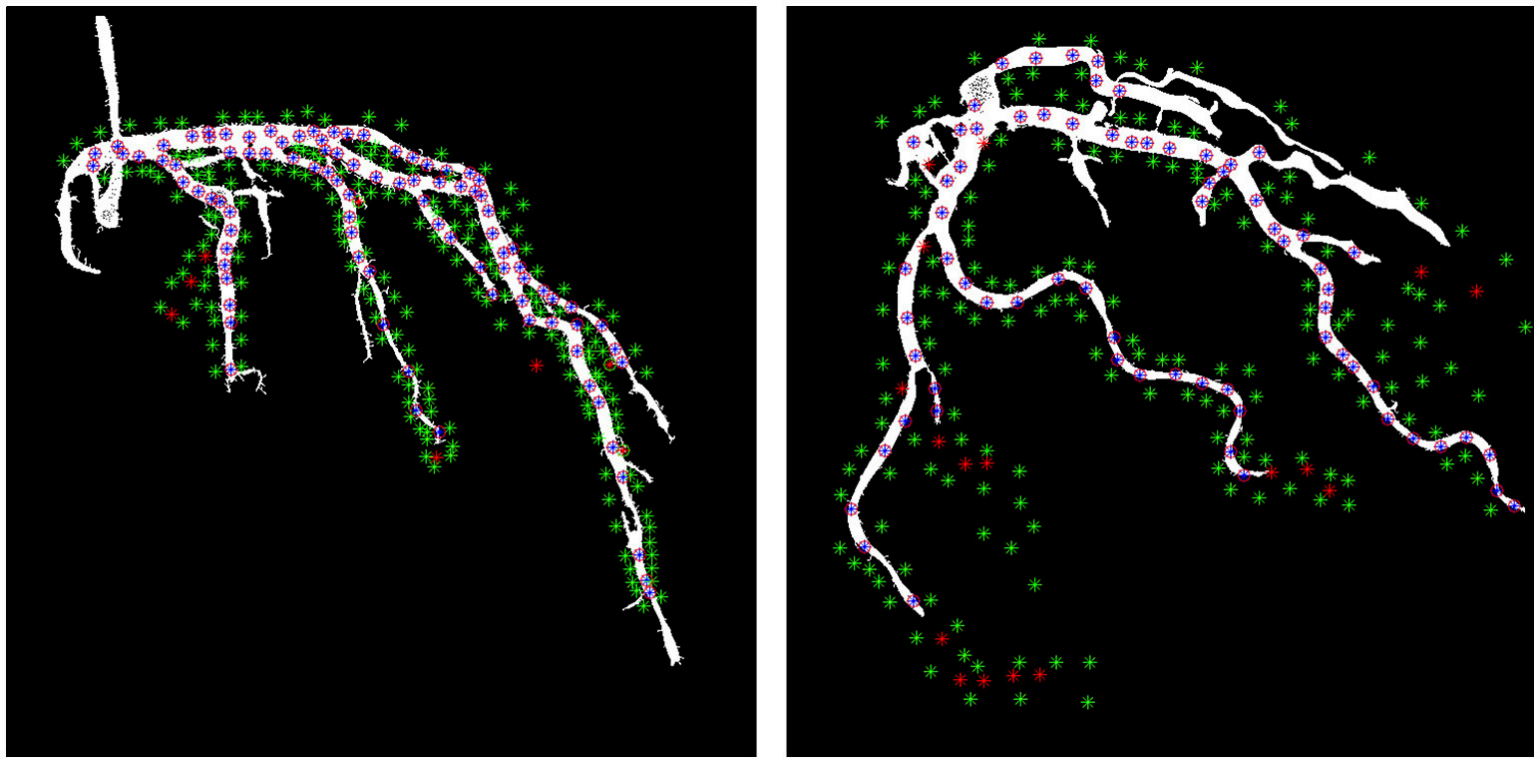
Image segmentation results in a sample image overlaid with the manual annotations. The blue stars with red circle around them are True Positive, the green stars are True Negative and red stars are False Negative and red starts with green circle around them are False Positive.

Considering the location of these markers in the automatic segmentation of the artery-tree: if the vessel markers were inside the segmentations then these were True Positive (TP); if background markers were inside the segmentations these were False Positives (FP); background markers outside the segmentation were True Negatives (TN); vessel markers outside the segmentation were False Negatives (FN). Furthermore sensitivity, specificity and accuracy were estimated as follows: $Sensitivity=\;\frac{TP}{TP+FN}$, $Specificity=\;\frac{TN}{TN+FP}$, and $Accuracy=\;\frac{TP+TN}{TP+FP+TN+FN}$. The approach for evaluation of the automatic segmentation algorithm is illustrated in Figure 3. In this figure the binary results of automatic segmentation of coronary artery-tree is shown with the manual annotations of the artery-tree and the background superimposed. In accordance to the automatic segmentation results, the manual annotations are divided into, (1) True Positives (blue stars enclosed with red circles) (2) True Negatives (green stars) (3) False Negatives (red stars) (4) False Positives (red stars enclosed with green circle).

## Results

**Figure 4:**
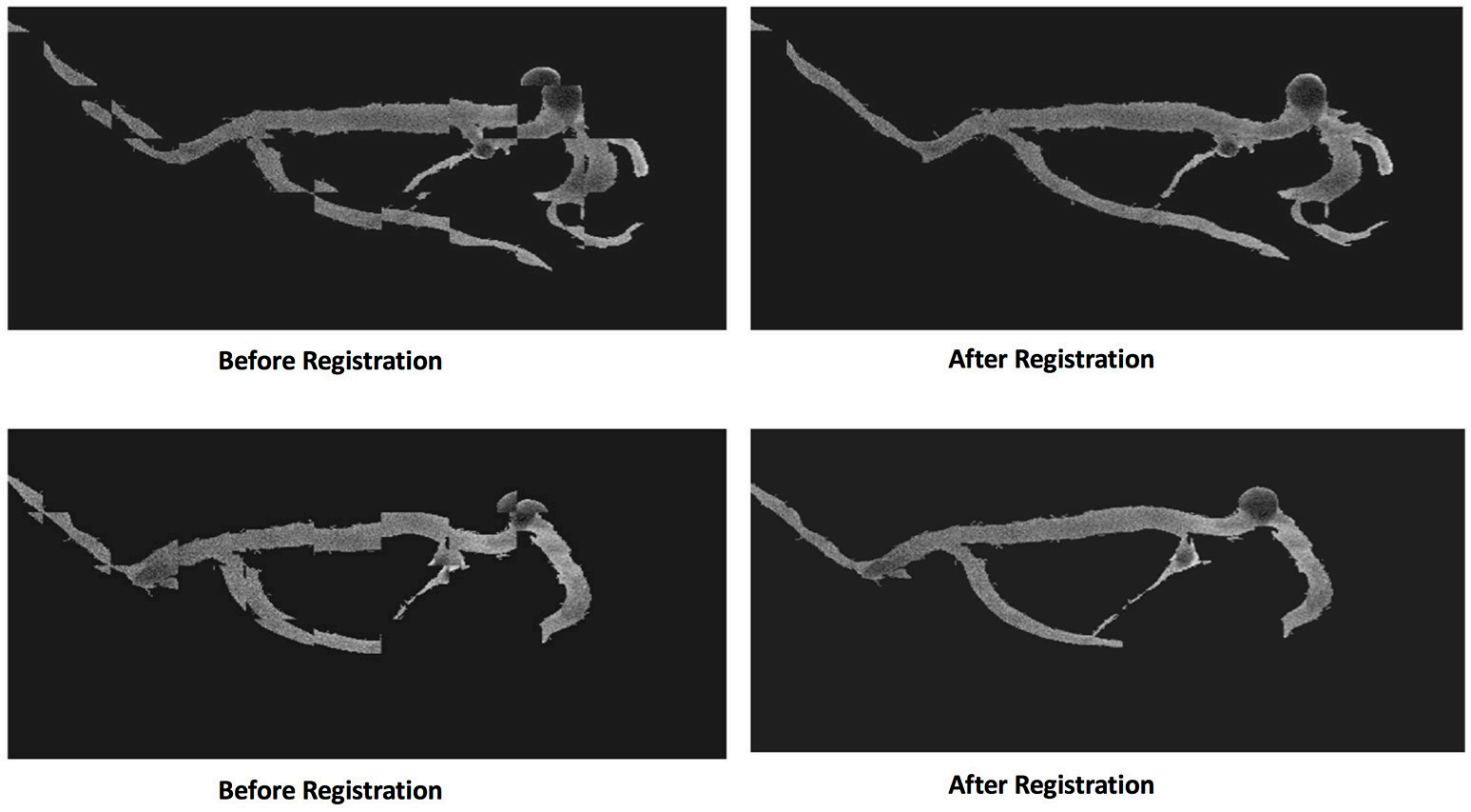
The effect of registration technique in two pairs of consecutive images. The chessboard is used to show the difference between two consecutive images before and after registration.

**Figure 5:**
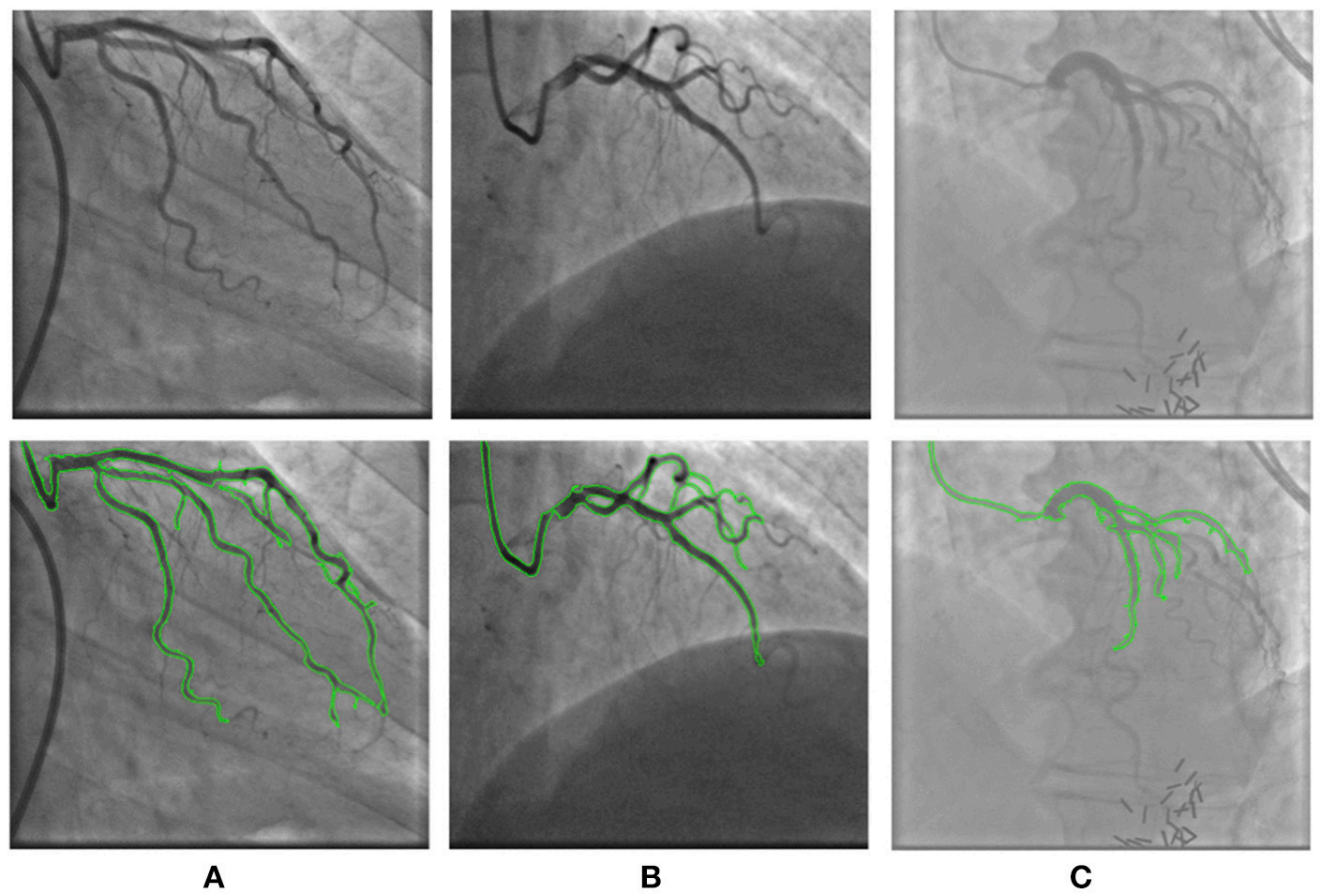
The results of segmentation in three sample images from different patients. First row shows the original images and second row shows the overlay of the original image with the boundary of the segmented artery-tree (green curves). The segmentation method overcame the artifacts and noise in **(A)** patient 2 and **(B)** patient 7, but in **(C)** patient 8, due to severe artifacts some parts of the artery-tree is missed.

The characteristics of the patients enrolled in this study are summarized in Table 1. An example illustrating the effect of the registration approach is shown in Figure 4 using the chessboard visualization technique, where the black pieces of the chessboard show the first image and the white pieces show the second image. This shows that the artery-trees are better aligned and change more smoothly after the registration. Figure 5 shows the results of segmentation algorithm in three sample images from different patients. In this figure the original images are shown with the outer boundary of the artery-tree (green curves) superimposed. The results of subjective evaluation of the processed images for 11 randomly selected patients show accuracy of 97%, specificity of 99%, and sensitivity of 93%.

**Table 1:** Data are presented as n(%) and mean±*SD*.

**Total patients**	**21**
Average age, y	59.4 ± 8.4
**Sex**	
Male	10(47.6)
Female	11(52.4)
Height (cm)	173 ± 10
Weight (kg)	84.4 ± 14.5
**Smoker (incl. previous)**	
Yes	10 (47.7)
No	7 (33.3)
Unknown	4 (19)
**Previous history**	
Hypertension	10 (47.6)
Atrial fibrillation	0 (0)
Diabetes Mellitus	3 (14.3)
Cerebral ischemia/TIA	0 (0)
Peripheral vascular disease	0 (0)
**Medications**	
ACE-I	0 (0)
ARB	8 (38.1)
Beta blocker	5 (23.8)
Calcium blocker	2 (9.5)
ASA	9 (42.9)
Clopidogrel	0 (0)
Statin	10 (47.6)
Nitrate	2 (9.5)

*TIA, transient ischemic attack; ACE-I, angiotensin-converting-enzyme inhibitor; ARB, Angiotensin II receptor blockers; ASA, Acetylsalicylic acid*.

**Figure 6:**
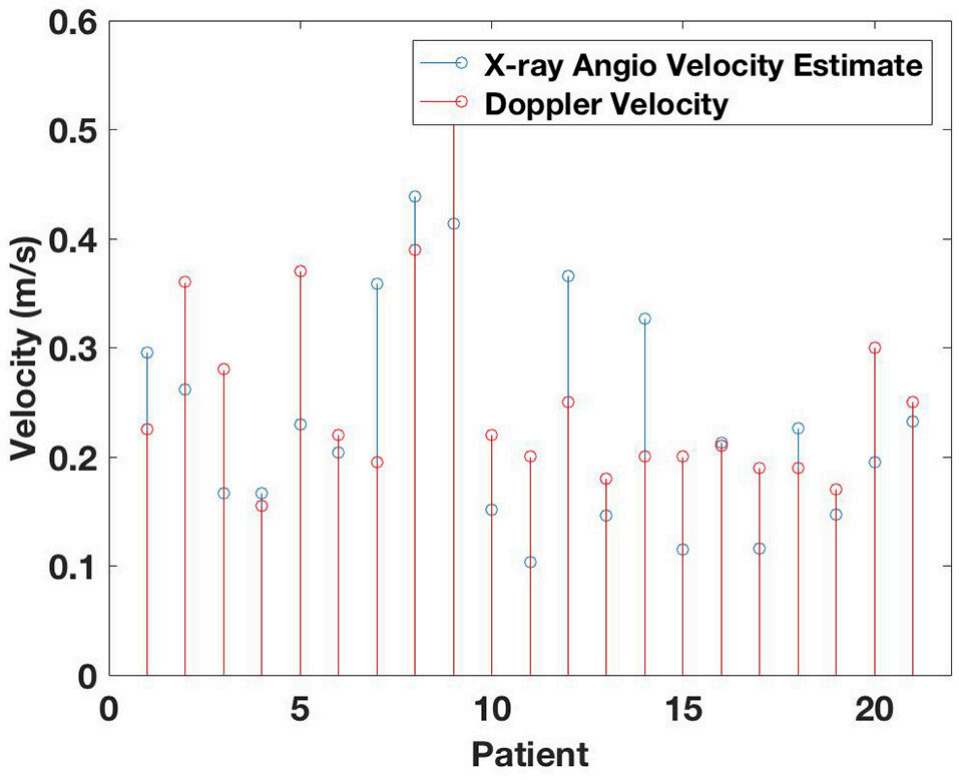
Estimated velocity from proposed method in comparison to the measured velocity using Doppler transthoracic imaging.

The estimated velocity for all the patients using the proposed multi-step algorithm on the X-ray images is illustrated in Figure 6, showing a good relation between the two measured velocities. Table 2 shows the velocity estimation from *Mlength*, as well as the numbers from *Mslope* in comparison to the measured velocity in LAD using transthoracic Doppler imaging, in addition to the correlation results using Spearman and Pearson tests. The one-sample *K*-test verified normal distribution of the velocity measurements (*p* < 0.05). We found a moderate but significant correlation between flow velocity assessed by Doppler and the proposed *Mlength* method: (*r*_*s*_ = 0.55, *p* < 0.008 Spearman, *r*_*p*_ = 0.58, *p* < 0.005 Pearson). Similar correlation and significance were found for the *Mslope* method: (*r*_*s*_ = 0.50, *p* < 0.02 Spearman, *r*_*p*_ = 0.55, *p* < 0.009 Pearson).

**Table 2:** Estimated slopes from proposed method *Mslope*, and estimated velocity from proposed method, *Mlength*, in comparison to the measured velocity using Doppler transthoracic imaging.

	**Patient no**.	***Mslope***	***Mlength* [m/s]**	**Doppler [m/s]**
	1	3.12	0.29	0.22
	2	1.18	0.26	0.36
	3	1.47	0.16	0.28
	4	2.34	0.16	0.15
	5	3.08	0.22	0.37
	6	1.64	0.20	0.22
	7	2.06	0.35	0.19
	8	5.48	0.43	0.39
	9	3.66	0.41	0.52
	10	0.79	0.15	0.22
	11	0.37	0.10	0.20
	12	3.05	0.36	0.25
	13	0.87	0.14	0.18
	14	2.61	0.32	0.20
	15	1.01	0.11	0.20
	16	2.88	0.21	0.21
	17	0.97	0.11	0.19
	18	1.94	0.22	0.19
	19	1.29	0.14	0.17
	20	1.98	0.19	0.30
	21	2.56	0.23	0.25
Pearson test		*r* = 0.55 *p* = 0.009	*r* = 0.58 *p* = 0.005	
Spearman test		*r* = 0.50 *p* = 0.02	*r* = 0.55 *p* = 0.008	

*The numbers from Mslope are in (pixel area)/(frame time interval), equivalent to $\text{[}\frac{{\left(0.2\;mm\right)}^{2}}{0.066\;s}$] and should not be compared directly, but are expected to be correlated to velocity*.

## Discussion and Conclusion

The main finding in the current study is that coronary blood flow velocity estimated from cine X-ray angiographic sequences using a fully automatic novel method correlates moderately with velocities measured using the more conventional method of transthoracic Doppler.

Image processing techniques are used to initially segment the artery-tree from each frame in an X-ray angiographic sequence; thereafter the heart motion is modeled and removed using non-rigid image registration. Suppressing the effect of the heart motion provides the ability to use the information from the previous and following frames in the time sequence to improve the segmentation of the artery-tree. With the availability of the segmented artery tree for each frame in the time sequence during contrast fluid propagation, the blood flow velocity is estimated from the velocity of the contrast fluid propagation (*Mlength* method). Flow velocity measurements from contrast angiograms is previously performed in anesthetized cats and rabbits to describe the physiology of the pulmonary circulation, using a specially designed X-ray apparatus (25). As far as we know, calculations of coronary flow velocity based on the contrast fluid propagation employing standard coronary cine X-ray angiographic sequences in humans has not been done before.

The most important advantage of our proposed method is that it is based on selective coronary angiographic sequences (2D frames in a time sequence). Selective coronary angiography is still the routine procedure for obtaining anatomical information for clinical decision-making in patients presenting with suspected coronary artery disease and it is cost effective compared to other techniques.

This is a prospective observational study in patients admitted for coronary angiography due to angina pectoris. All patients had normal or near normal coronary arteries. The coronary flow velocity was assessed under controlled circumstances with Doppler. The angiography was performed in a clinical setting with all the angles and heights registered, but without standardized injection velocity and volumes. However, this might strengthen the method as a clinical approach. The contrast dye is injected by a well-trained interventional cardiologist and is expected not to seriously affect blood-flow velocity.

### Limitations

There are some limitations of the study.

1. The number of study samples is limited to 21 patients. The method is developed by studying and experimenting on the cine X-ray angiographic sequences of these 21 patients, thus it is necessary to validate the findings on a new and larger dataset.
2. Some assumptions are made when estimating the velocity. From Ass1 in the method section: we assume that all vessels in the area of interest, i.e., medium coronary arteries, are of constant Areal, *A*_*i*_ = *A*. This would be a limitation where the vessels become thinner further into the branches, but we are not concerned with these smallest arteries and capillaries.
3. The different views are chosen by the clinician during the procedure to maximize the view of the artery under consideration. This means that it is chosen to get the upper and mid part of the artery as tangential to the X-ray projection as possible. The method estimating the length of the vessels, *n*·Δ*L*, based on the skeletonization of the segmented vessels assumes the vessels to be tangential with the view, which is not always true, thus imposing a limitation. The velocity estimate is made over a number of frames from the start of the contrast propagation, *t*_*s*_, to the time of the first maxima of the polynomial function, *t*_*max*_, hoping that this would provide an averaging effect removing some of the noise caused by both the projection not being tangential but also to the segmentation having inaccuracies.
4. The velocity estimation relies heavily on the segmentation, thus mistakes and inaccuracies in the segmentation might lead to wrongly estimated velocities.
5. The segmentation results rely on the registration and modeling. Modeling the non-rigid movement of the heart in the existence of other rigid and non-rigid movements such as patient breathing and sudden body movement due to probable pain is not an easy task and sometimes its accuracy is affected. Moreover, non-rigid registration is a time-demanding procedure and the on-site estimation of blood flow velocity in real-time is yet not an option.

### Conclusion and Further Work

The main finding in the current study is that coronary blood flow velocity estimated from cine X-ray angiographic sequences using a fully automatic method are moderately correlated with velocities measured using conventional method of transthoracic Doppler. The method should be verified in a larger dataset.

The results show a moderate correlation, but are not as close to the measured velocity as one could hope. This can partly be caused by limitations 2, 3, 4, and 5. We will continue working on improving segmentation and registration, to deal with the effect of limitations 4 and 5. There are no simple solutions to limitations 2 and 3, but we are currently looking into the possibility of developing an approximate 3D reconstruction based on 3 or more selective coronary angiographic sequences (2D frames in a time sequence). For such a 3D reconstruction to be possible, landmark points have to be identified in the different sequences, in addition a synchronization with the heart beat sequence is necessary. Thus, the heart motion needs to be adequately modeled.

In future work we want to calculate the coronary flow reserve (CFR) for assessment of microcirculation as the etiology of angina in patients with normal coronary arteries. Further research will include X-ray angiography sequences after infusion of a vasodilator (Adenosine), in addition to the baseline as in the present study. Adenosine is a natural occurring substance in the body, and interventional cardiologists utilize adenosine during such procedures to maximally increase the blood flow by reducing the resistance in the microvessels. There is a potential for assessing the microvascular function by using the ratio of basal and adenosine stimulated blood velocity estimations as an estimate of CFR. Some few approaches have been presented to assess CFR by using CT angiography, (12) and (11). However, information concerning flow changes with the cardiac cycle is lacking. In the proposed method, the area of the coronary artery-tree covered by the contrast dye is modeled by a 7° polynomial, capturing the cardiac cycles with the periodic changes in the shape of the coronary arteries over time. This information helps estimating the time duration for the first start-to-complete cycle of propagation of the contrast dye injection.

For potential real-time purposes in the future, powerful computational tools are needed for the means of non-rigid image registration.

## Author Contributions

MK proposed the methodology and carried out all analyses and wrote the first draft of the manuscript. AIL formulated the problem of estimating the blood flow velocity from X-ray angiograms. KE and TE provided input regarding the image analyses and velocity estimation methodology. KE provided major inputs on the revision of the manuscript. AIL and CS provided the datasets and input on data acquisition and patient enrollment. MK, KE, AIL, TE and CS jointly wrote the final version of the manuscript.

### Conflict of Interest Statement

The authors declare that the research was conducted in the absence of any commercial or financial relationships that could be construed as a potential conflict of interest.
